# Bootstrap percolation on spatial networks

**DOI:** 10.1038/srep14662

**Published:** 2015-10-01

**Authors:** Jian Gao, Tao Zhou, Yanqing Hu

**Affiliations:** 1 CompleX Lab, Web Sciences Center, University of Electronic Science and Technology of China, Chengdu 611731, China; 2 Big Data Research Center, University of Electronic Science and Technology of China, Chengdu 611731, China; 3 School of Mathematics, Southwest Jiaotong University, Chengdu 610031, China; 4 School of Information Science and Technology, Sun Yat-sen University, Guangzhou 510006, China

## Abstract

Bootstrap percolation is a general representation of some networked activation process, which has found applications in explaining many important social phenomena, such as the propagation of information. Inspired by some recent findings on spatial structure of online social networks, here we study bootstrap percolation on undirected spatial networks, with the probability density function of long-range links’ lengths being a power law with tunable exponent. Setting the size of the giant active component as the order parameter, we find a parameter-dependent critical value for the power-law exponent, above which there is a double phase transition, mixed of a second-order phase transition and a hybrid phase transition with two varying critical points, otherwise there is only a second-order phase transition. We further find a parameter-independent critical value around −1, about which the two critical points for the double phase transition are almost constant. To our surprise, this critical value −1 is just equal or very close to the values of many real online social networks, including LiveJournal, HP Labs email network, Belgian mobile phone network, etc. This work helps us in better understanding the self-organization of spatial structure of online social networks, in terms of the effective function for information spreading.

Bootstrap percolation was originally introduced by Chalupa, Leath and Reich^1^ in the context of magnetic disordered systems in 1979. Since then, it has been studied extensively by physicists and sociologists, mainly due to its connections with various physical models and a variety of applications such as neuronal activity^2^ and jamming transitions^3^. Bootstrap percolation can be essentially considered as an activation process on networks: (i) Nodes are either active or inactive; (ii) Once activated, a node remains active forever; (iii) Initially, each node is in an active state with a given probability *p*; (iv) Subsequently, inactive nodes become active if they have at least *k* active neighbors; (v) Nodes are activated in an iterative manner according to the condition in (iv), until no more nodes can be activated. This process has been investigated on different kinds of networks including lattices^4^,^5^, trees^6^,^7^, random networks^8^,^9^,^10^,^11^, and so on.

Bootstrap percolation has found applications in explaining many important social phenomena, such as the spreading of information^12^, the propagation of infection^13^,^14^, the adoption of new products and social behaviors^15^,^16^,^17^,^18^ including trends, fads, political opinions, belief, rumors, innovations and financial decisions. For instance, in emergence of cultural fads and adoption of new technology or objects, an individual can be positively influenced when there is a sufficient number of its close friends who have also done so^19^. In other words, one may decide to buy a product when recommended by at least *k* users and trust a message when told by at least *k* neighbors; cf. the well-known rule, “What I tell you three times is true”^20^. In this way, the process leads initially localized effects propagating throughout the whole network. Moreover, a broad range of generalized formulations of bootstrap percolation on social networks are investigated, such as Watts’ model of opinions^21^, in which *k* is replaced by a certain fraction of the neighbors, and disease transmission models with different degrees of severity of infection^22^.

Real networks are often embedded in space^23^ and social networks are no exception. Previous empirical studies of online social networks^24^, email networks^25^ and mobile phone communication networks^26^ have confirmed a spatial scaling law, namely, the probability density function (PDF) of an individual to have a friend at distance *r* scales as 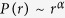, 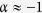
27. In fact, prior to these empirical observations, Kleinberg^28^ has proposed a spatial network model by adding long-range links to a 2-dimensional lattice, and he has proved that when 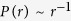, the structure is optimal for navigation. Recently, Hu *et al.*^27^ suggested the optimization of information collection as a possible explanation for the origin of this spatial scaling law.

Extensive studies have shown that the spatial organization can change the dimension^29^,^30^,^31^,^32^,^33^, which dominates many important physical properties of networks^34^,^35^,^36^,^37^,^38^,^39^,^40^,^41^. Moukarzel *et al.*^42^ studied k-core percolation on long-range spatial networks, which is built by taking a 2-dimensional lattice and adding to each node one or more long-range links. In the networks, the probability density function (PDF) of long-range links with length *r* scales as 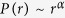. By numerical simulation, they found that the 3-core transition is of first-order for *α* > −1.75 (it is equivalent to the scaling of 2.75 found by Moukarzel *et al.*^42^) and of second-order for smaller *α*. In fact, k-core percolation has close relation to bootstrap percolation^43^,^44^, nevertheless the two processes have different features from each other, being strongly dependent on the network structure^8^,^45^. Although there is a deeper understanding of percolation processes and spatial networks now, how spatial organization influences the spreading process on social networks under the framework of bootstrap percolation remains further investigation.

In this paper, we numerically study bootstrap percolation on undirected Kleinberg’s spatial networks, which is a typical artificial social network. Setting the relative size of the giant active component to the network size as the order parameter, we find that the distribution of long-range links’ lengths can change the order of phase transition. In particular, our main findings are as follows: (i) We find a parameter-dependent critical value *α*_*c*_, above which a double phase transition^46^ is observed. Here, the so-called double phase transition means a mixture of two transitions at different critical points, consisting of a hybrid phase transition and a second-order one. In this paper, we use the hybrid phase transition to indicate a first-order phase transition in which the order parameter has a discontinuous jump between two non-zero values. (ii) Surprisingly, we find a parameter-independent critical value 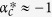, about which the two critical points for the double phase transition are almost constant. (iii) When 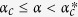, the first-order critical point decreases and the second-order critical point increases as *α* decreases. When *α* < *α*_*c*_, there is only a second-order phase transition with an increasing critical point as the decreasing of *α*. Furthermore, we test the universality of our findings by drawing the phase diagram and give a possible explanation of the rich phase transition phenomena by simulating on related networks. Our findings indicate that the spatial scaling 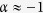, observed in real social networks, may be resulted from some deep-going principles in addition to the optimization of navigation and information collection, which is not yet fully understood now.

## Results

**Figure 1:**
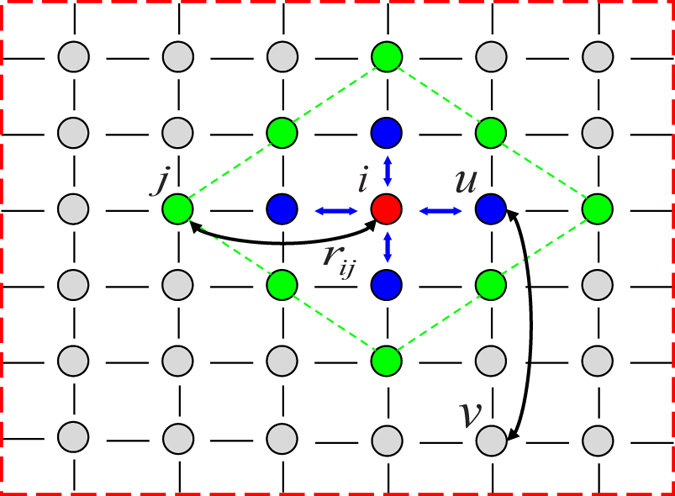
Illustration of the undirected Kleinberg’s spatial network constrained on a 2-dimensional periodic square lattice. Each node has four short-range links (colored blue for node *i*) and one long-range link (colored black). The probability density function (PDF) of a node to have a long-range link at Manhattan distance *r* scales as 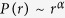. For the target node *i* (colored red), when *r* = 2, there are eight candidate nodes (colored green), from which we can choose an uncoupled node *j* to make a connection. For another target node *u*, we can choose to connect it with node *v* when *r* = 3.

Kleinberg model^28^ is a typical spatial network model, which has been well justified by empirical data^24^,^25^,^26^. Here, the undirected Kleinberg’s spatial network is constrained on a 2-dimensional periodic square lattice consisting of *N* = *L* × *L* nodes. In addition to its initially connected four nearest neighbors, each node *i* has a random long-range link to a node *j* with probability 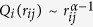, where *α* is a tunable exponent and *r*_*ij*_ denotes the Manhattan distance, which quantifies the length of the shortest path between node *i* and node *j*, following strictly the horizontal and/or vertical links in lattices. Since the number of nodes at distance *r* to a given node is proportional to *r*^*d−*1^ in a *d*-dimensional lattice, the probability *Q*(*r*_*ij*_) can be mapped to a probability density function (PDF), 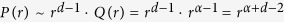. In the present 2-dimensional case, where *d* = 2, the probability density function(PDF) scales as 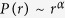. An illustration of a 2-dimensional undirected Kleinberg’s spatial network can be found in Fig. 1.

In the following, we focus on three indicators: (i) The relative size of the giant active component (*S*_*gc*_) at the equilibrium, i.e., the probability that an randomly selected node belongs to the giant active component; (ii) The number of iterations (*NOI*) to reach the equilibrium, which is usually used to determine the critical point for the first-order phase transition^47^,^48^; (iii) The relative size of the second giant active component (*S*_*gc*2_), which is usually used to determine the critical point for the second-order phase transition^32^,^48^.

**Figure 2:**
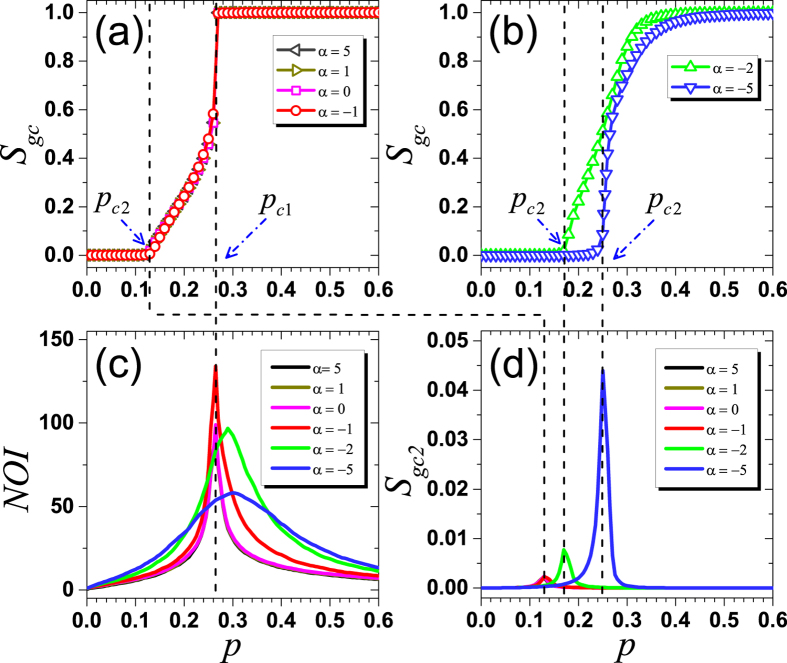
*S*_*gc*_, *NOI* and *S*_*gc*2_ as a function of *p* for different *α* after *k* = 3 bootstrap percolation on undirected Kleinberg’s spatial networks. Two different types of *S*_*gc*_(*p*) curves are observed, including a double phase transition (**a**) and a second-order one (**b**). When *α* ≥ −1, *S*_*gc*_(*p*) curves behave alike and a double phase transition is present. *S*_*gc*_ abruptly jumps to 1 at 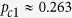, where *NOI* reaches its maximum (**c**). When *α* < −1, there is only a second-order phase transition with an increasing critical point as the decreasing of *α*, where *S*_*gc*2_ reaches its maximum at different *p*_*c*2_ (**d**). Dash lines mark identification of critical points. Results are obtained by simulations on networks with fixed size *L* = 400 and averaged over 1000 realizations.

Figure 2 shows rich phase transition phenomena when taking *S*_*gc*_ as the order parameter. When *α* ≥ −1, the curves of *S*_*gc*_(*p*) are well overlapped and the system undergoes a double phase transition, mixed of a hybrid phase transition and a second-order one as shown in Fig. 2a. Notice that *S*_*gc*_ has a continuous increase at 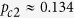 (the second-order critical point), where the transition is of second-order. In contrast, *S*_*gc*_ has an abrupt jump directly from around 0.58 to almost 1 at 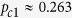 (the first-order critical point), where there is a hybrid phase transition. Surprisingly, the two critical points seem to be constant when 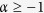, as indicated by the four overlapped *S*_*gc*_(*p*) curves in Fig. 2a. When *α* < −1, there is only a second-order phase transition with an increasing *p*_*c*2_ as the decreasing of *α* (see Fig. 2b). Specifically, 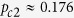 when *α* = −2 and 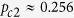 when *α* = −5. Although *S*_*gc*_ goes up sharper after *p* exceeds *p*_*c*2_ as *α* getting smaller, simulations justify that the curve of *S*_*gc*_(*p*) is still continuous, meaning that the transition is indeed of second-order when *α* < −1.

Finding critical points via simulations is always a difficult task that requires high precision. When *α* ≥ −1, where a part of the double phase transition is a hybrid phase transition, we can determine the critical point *p*_*c*1_ by calculating the number of iterations (*NOI*) in the cascading process, since *NOI* sharply increases when *p* approaches *p*_*c*1_ for the first-order phase transitions^47^,^48^. Accordingly, *p*_*c*1_ is calculated by plotting *NOI* as a function of *p*. As shown in Fig. 2c, *NOI* reaches its maximum at the same *p* when *α* ≥ −1, which is the evidence that 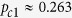 is almost a constant value. Analogously, by plotting *S*_*gc*2_ as a function of *p*, we can precisely identify *p*_*c*2_^32^,^48^, at which *S*_*gc*2_ reaches its maximum (see Fig. 2d). We can see that *p*_*c*2_ increases as *α* decreases, as 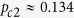 (*α* ≥ −1), 0.176 (*α* = −2) and 0.256 (*α* = −5).

**Figure 3:**
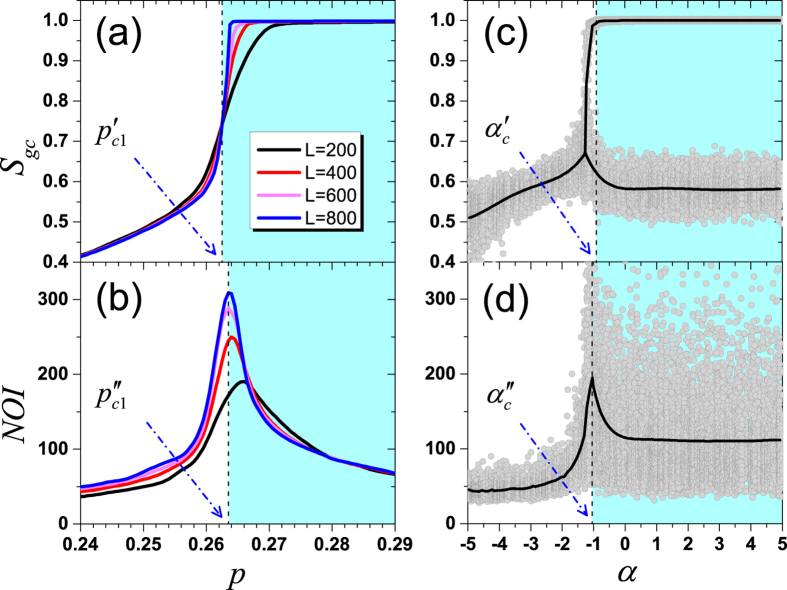
Cross-validation of *p*_*c*1_ and *α*_*c*_. (**a**,**b**) are for *S*_*gc*_ and *NOI* under different network size *L* when *α* = −1, respectively. There is an intersection of *S*_*gc*_(*p*) at 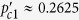, while *NOI* reaches its maximum at 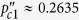 when *L* = 800. Thus, *p*_*c*1_ is identified as the average value 0.263. (**c**,**d**) are for *S*_*gc*_ and *NOI* when *p* = 0.263 under different *α*, respectively. For 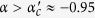, *S*_*gc*_ has two phases, and *NOI* reaches its maximum at 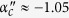. Thus, *α*_*c*_ is identified as the average value −1. In (**c**,**d**), dark curves respectively represent the average values of *S*_*gc*_ and *NOI*, obtained from 1000 realizations, and each data point stands for one realization.

Although to justify the hybrid phase transition and to determine the critical value *α*_*c*_ by simulations in a finite discrete system are not easy, we solve this problem by a cross-validation on the critical point *p*_*c*1_ and the critical value *α*_*c*_. Firstly, we fix *α* = −1 to determine *p*_*c*1_. On the one hand, there is an intersection for curves of *S*_*gc*_(*p*) at 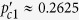 under different network sizes as shown in Fig. 3a, which can be considered as the critical point according to the finite-size analysis^48^. On the other hand, the corresponding *NOI* reaches its maximum at 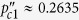 when *L* = 800 as shown in Fig. 3b. Combining these two observations, a more appropriate critical point is identified as the average value 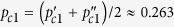. Conversely, we fix *p* = 0.263 to determine *α*_*c*_. From Fig. 3c, we can see that *S*_*gc*_ has two phases: about 0.58 or close to 1 when 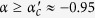, which is a strong evidence that *S*_*gc*_ undergoes a hybrid phase transition. If the increasing of *S*_*gc*_ is continuous, there is no such gap between the two phases. From Fig. 3d, we note that the corresponding averaging *NOI* reaches its maximum at 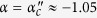. Combining these two evidences, we appropriately identify the critical value as the average value 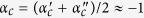.

**Figure 4:**
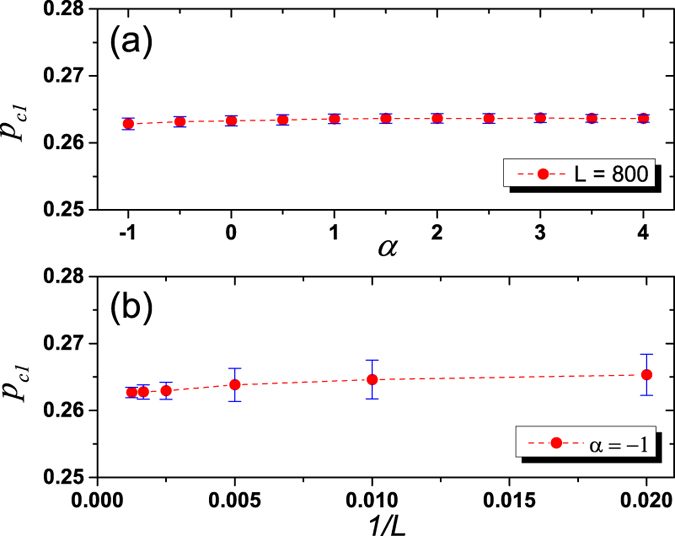
Error estimation and effects of finite-size. (**a**) Error estimation of *p*_*c*1_ with fixed network size *L* = 800. As *α* approaches the critical value −1, *p*_*c*1_ slightly decreases, but being kept in a very narrow range. When *α* is in range [−1,4], the mean value of *p*_*c*1_ is 0.2634, and the difference between maximum and minimum value of *p*_*c*1_ is 0.0008. (**b**) Analysis on the effects of finite-size. The data point and the error bar are respectively mean value and standard deviation of the identified *p*_*c*1_ in the case of *α* = −1 under different network sizes *L*. As *L* goes to infinity (from the right side to the left side in (**b**)), the mean value of *p*_*c*1_ gradually approaches an extreme value around 0.263, and the standard deviation of the *p*_*c*1_ decreases. Results are obtained by averaging over 1000 realizations.

In addition, *p*_*c*1_ for the hybrid phase transition should be almost constant when *α* ≥ *α*_*c*_, otherwise we cannot observe the separation of two phases in Fig. 3c for a fixed value *p* = 0.263. To verify that, we estimate errors of *p*_*c*1_ by varying *α* under fixed network size *L*. As shown in Fig. 4a, *p*_*c*1_ slightly decreases when *α* approaches the critical value 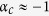. Even though, when *α* is in the range [−1,4], the difference between maximum and minimum value of *p*_*c*1_ is only 0.0008 after over 1000 realizations, which is very small compared to the whole range of *p* (i.e. [0,1]), indicating that the value of *p*_*c*1_ is not sensitive to the parameter *α* when *α* ≥ −1. Based on these evidences, *p*_*c*1_ can be roughly considered as a constant and its value is about 0.2634, which is the mean value of *p*_*c*1_ when *α* in the range [−1,4]. Furthermore, taking *α* = −1 as an example, we consider the effects of finite-size of networks on these results. As shown in Fig. 4b, the mean value of *p*_*c*1_ gradually approaches an extreme value around 0.263, and the standard deviation of *p*_*c*1_ decreases as *L* goes to infinity. Similar results hold for the analysis of the critical point *p*_*c*2_ and its value is also almost constant as 0.134.

**Figure 5:**
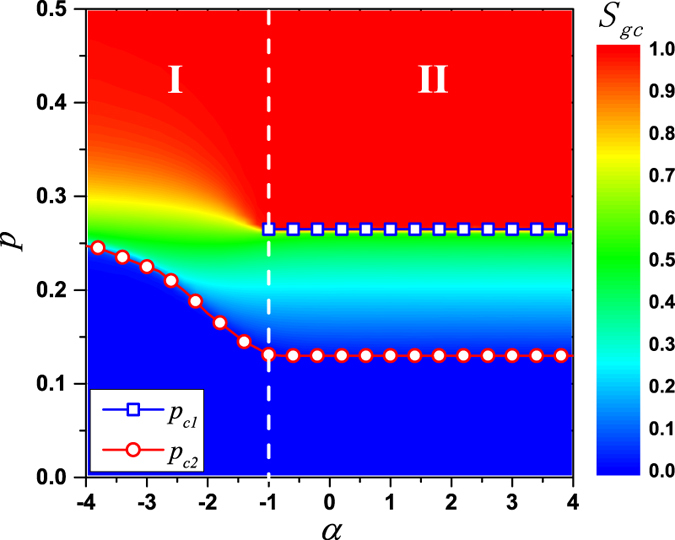
Phase diagram of *k* = 3 bootstrap percolation in the *p* − *α* plane. The color marks the value of *S*_*gc*_. Dashed lines with solid squares and circles represent *p*_*c*1_ and *p*_*c*2_, respectively. Separated by *α* = −1, a double phase transition is observed in the right region II with two almost constant critical points 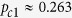 and 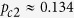. In the left region I, only a second-order phase transition is present with an increasing critical point as *α* decreases. The critical point is up to 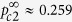, which is obtained in the case of all long-range links’ lengths are 2. Results are averaged over 1000 realizations with fixed network size *L* = 400.

A representative phase diagram for *S*_*gc*_ in the *p* − *α* plane is shown in Fig. 5. We find that the varying of *α*, which dominates the distribution of long-range links’ lengths, can change the order of phase transition. Overall, 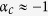 is confirmed to be a critical value, above which a double phase transition (region II) is present. When *α* ≥ −1, the curves of *S*_*gc*_(*p*) are overlapped, suggesting that the properties of bootstrap percolation on these spatial networks are alike. When *α* < −1, the hybrid phase transition vanishes and *S*_*gc*_ only undergoes a second-order phase transition (region I) with an increasing critical point as the decreasing of *α*. The maximum of *p*_*c*2_ is about 0.259, which is obtained when 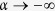, i.e., all long-range links’ lengths are 2.

**Figure 6:**
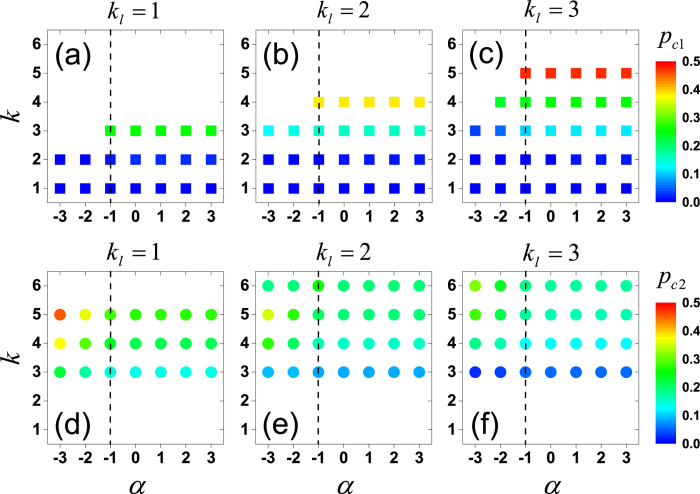
Phase diagram of bootstrap percolation on undirected Kleinberg’s spatial networks in parameter spaces (*k*, *α*, *k*_*l*_). The color of data points in (**a**–**c**) marks the value of *p*_*c*1_, where there is a hybrid phase transition (or a first-order phase transition in the trivial cases where 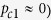, and the color of data points in (**d**–**f**) marks the value of *p*_*c*2_, where the transition is of second-order. Blank areas stand for the absent of the corresponding phase transitions. Separated by the vertical dash line *α* = −1, on the right side, the color of data points is nearly unchanged for the same parameter *k*, meaning that the values of *p*_*c*1_ and *p*_*c*2_ are almost invariant. 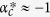 is found to be a parameter-independent critical value, above which the critical points for the double phase transition are almost constant. When 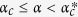, *p*_*c*1_ decreases and *p*_*c*2_ increases as *α* decreases. When *α* < *α*_*c*_, *p*_*c*2_ increases as *α* decreases. Results are averaged over 1000 realizations with fixed network size *L* = 400.

To test the universality of the findings, we simulated on undirected Kleinberg’s spatial networks in parameter spaces (*k*, *α*, *k*_*l*_) and determined the critical points. Results are shown in Fig. 6. According to the relationship between the threshold *k* and half of the average degree of the network 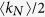, where 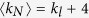, there are three regions in the phase diagram:

- When *k* is remarkably smaller than 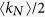, e.g, *k* = 1 compared to 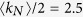, there is only a trivial first-order phase transition at 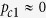.
- When *k* is around 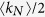, e.g., *k* = 3 compared to 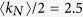, there is a critical value *α*_*c*_, above which a double phase transition is observed. The value of *α*_*c*_ depends on the choice of both *k* and *k*_*l*_. In particular, 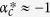 is found to be a parameter-independent critical value, about which the two critical points for the double phase transition are almost constant. Specifically, as shown in the phase diagram of Fig. 6, the color of data points for the same parameter *k* is nearly unchanged when *α* ≥ −1, which is a strong evidence that the values of *p*_*c*1_ and *p*_*c*2_ are almost constant. When 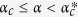, *p*_*c*1_ decreases and *p*_*c*2_ increases as *α* decreases. Note that *α*_*c*_ can be equal to 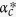 in some parameter spaces, such as 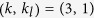 and 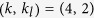.
- When *k* is remarkably larger than 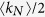, e.g, *k* = 5 compared to 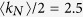, the hybrid phase transition is absent and *S*_*gc*_ only undergoes a second-order phase transition with an increasing *p*_*c*2_ as the decreasing of *α* (see Supplementary Figs S1-S3 for the detailed shapes of *S*_*gc*_(*p*) curves).

Moreover, simulations confirm that our main results also hold for Kleinberg’s spatial networks with directed long-range links since 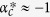 is still a critical value. However, there is only a first-order phase transition with *p*_*c*1_ being almost constant instead of the formal double transition when *α* ≥ −1 (see Supplementary Fig. S4). In addition, simulations on undirected Kleinberg’s spatial networks without periodic boundary conditions suggest that whether the square lattice has periodic boundary conditions does not essentially affect our main results (see Supplementary Fig. S5).

**Figure 7:**
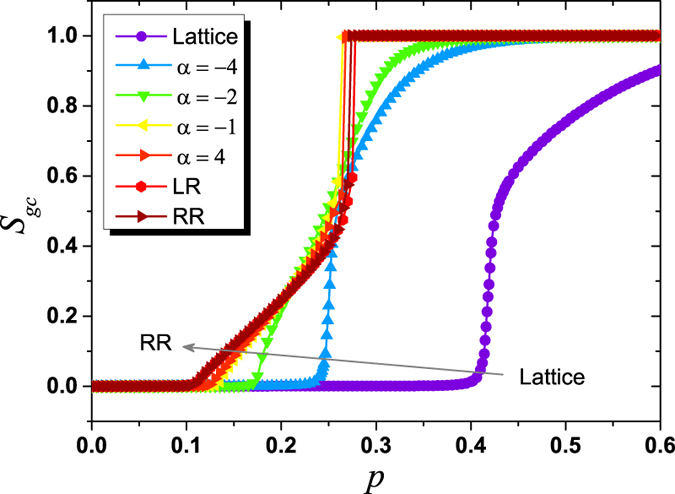
*S*_*gc*_ as a function of *P* after *k* = 3 bootstrap percolation on different networks, including Lattice, LR, RR and the present spatial networks with different *α*. When *α* = −4, the curve of *S*_*gc*_(*p*) on the spatial network behaves like the one on Lattice network, and the transition is of second-order. As *α* increases, the transition turns into a double phase transition when *α* ≥ −1, where the curves of *S*_*gc*_(*p*) are almost overlapped with the ones on LR network and RR network. The parameter for LR network is set as *α* = −1. Results are averaged over 1000 realizations with fixed network size *L* = 400.

To provide the insights on the mechanism of the transition, we simulate on different networks and compare with other related transitions. These networks include a simple 2-dimensional lattice (Lattice), networks with all 5 links being long-range (LR) and networks without spatial structure, i.e. random 5-regular networks (RR). In the LR network, which is a special case of long-range percolation model in the 2-dimensional space^49^,^50^,^51^, each node is associated with only *k*_*l*_ = 5 undirected long-range links instead of initially connected short-range links based on a 2-dimensional periodic lattice. As shown in Fig. 7, the curves of *S*_*gc*_(*p*) on the spatial networks are between the ones on Lattice network and RR network. When *α* = −4, the *S*_*gc*_(*p*) curve on the spatial network has similar trend with the one on Lattice network since the very long-range links are rare, and the transition is of second-order. When *α* ≥ −1, there is a double phase transition and the curves of *S*_*gc*_(*p*) are almost overlapped with the one on RR network. These observations indicate that, to turn the value of *α*, we can change the bootstrap percolation properties of spatial networks from Lattice network to RR network, or vice versa. More specifically, when *α* = −4, all long-range links are highly localized and the structure of spatial networks is similar to Lattice network, whereas when *α* ≥ −1, mainly due to the existence of very long-range links, the spatial networks behave like RR network.

**Figure 8:**
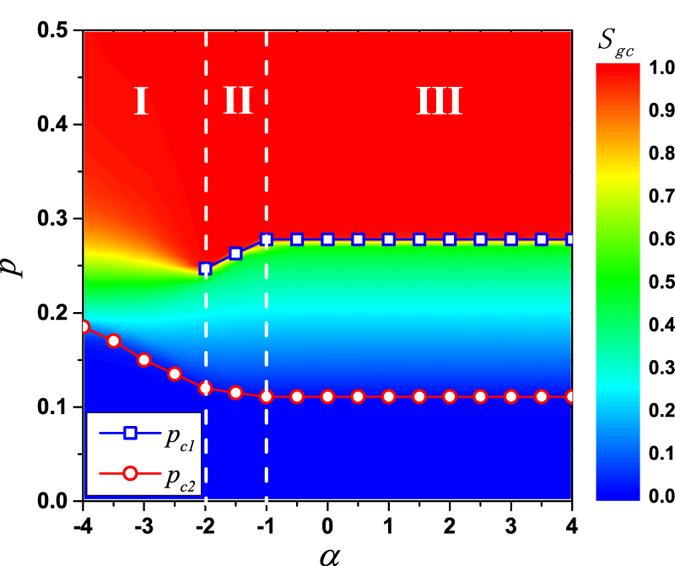
Phase diagram of *k* = 3 bootstrap percolation in the *p *−* α* plane on LR networks with *k*_*l*_ = 5. The color marks the value of *S*_*gc*_. When *α* decreases, the transition is of second-order with an increasing *p*_*c*2_ in the region I where *α* < −2. When *α* decreases, a double phase transition is observed in the region II where −2 ≤ *α* < −1, with a decreasing *p*_*c*1_ for the hybrid phase transition and an increasing *p*_*c*2_ for the second-order phase transition. In the region III where *α* ≥ −1, the double phase transition has two almost constant critical points, *p*_*c*1_ = 0.278 and *p*_*c*2_ = 0.111. Results are averaged over 1000 realizations with fixed network size *L* = 400.

Together, it should be noted that the *S*_*gc*_(*p*) curve on LR network when *α* = −1 acts like the ones on RR network and spatial networks when *α* ≥ −1. To better understand how does *α* affect the transition on LR network, taking *k*_*l*_ = 5 as an example, we show the phase diagram after *k* = 3 bootstrap percolation in Fig. 8. The diagram is divided into three regions by critical values 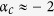 and 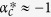. As *α* decreases, the transition is of second-order with an increasing *p*_*c*2_ when 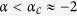 (region I). There is a double phase transition when 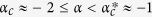 (region II), where *p*_*c*1_ decreases and *p*_*c*2_ increases as *α* decreases. Once again, a double phase transition with two almost constant critical points, 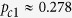 and 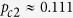, is observed when 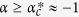 (region III). Further simulations suggest that similar main results also hold under other combinations of *k* and *k*_*l*_ (see Supplementary Fig. S6 for the phase diagram and Supplementary Figs S7–S11 for the shapes of *S*_*gc*_(*p*) curves).

In fact, for the original Kleinberg’s spatial networks with directed long-range links, Sen *et al.*^52^ found that the varying of *α* can change the network structure, namely, the network is regular-lattice-like when *α* < −2, small-world-like when −2 < *α* < −1 and random-like when *α *>−1. More recent studies^30^,^31^,^32^ also proposed three regimes: (i) When *α* > −1, the dimension of the spatial network is 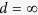 and the percolation transition belongs to the university class of percolation in Erdös-Rényi networks. (ii) When 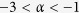, *d* decreases continuously from 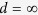 to *d* = 2 and the percolation shows new intermediate behavior. (iii) When *α* < −3, the dimension is *d* = 2 and the percolation transition belongs to the university class of percolation in regular lattices. These previous findings suggest that the properties of spatial networks have qualitative changes when *α* is around −1, which is corresponding to the observation of these phase transition phenomena here.

## Discussion

In summary, we have studied bootstrap percolation on spatial networks, where the distribution patterns of long-range links’ lengths can change the order of phase transition. In particular, we find a parameter-dependent critical value *α*_*c*_, above which a double phase transition, mixed of a hybrid phase transition at a higher *p* and a second-order phase transition at a lower *p*, is present. It is particularly interesting that we find a almost parameter-independent critical value 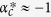, about which the curves of *S*_*gc*_(*p*) are well overlapped, indicating that the two critical points for the double phase transition are almost constant when *α* ≥ −1. As bootstrap percolation has not been studied yet on undirected Kleinberg’s spatial networks, our novel findings indicate that the topological properties of undirected Kleinberg’s spatial networks are alike when *α* ≥ −1 in the 2-dimensional space. In fact, the scaling law 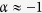 has been empirically observed in many real networks^24^,^25^,^26^, which may be resulted from complex self-organizing processes toward optimal structures for information collection^27^ and/or navigation^28^. Since the cascading processes on spatial networks are almost the same when *α* ≥ −1, 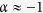 is indeed corresponding to the structure with the smallest average geographical length of links, which can exhibit as effective spreading of information as networks with even longer shortcut links. This is to some extent relevant to the principle of least effort in human behavior^53^.

We find the varying of *α* can change the bootstrap percolation critical behavior from random regular networks to lattices, or vice versa. In particular, when 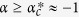, the spatial networks behave like random regular networks and there is a double phase transition. When 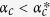, there is a richer phase transition phenomena. More specifically, the double phase transition is still present when 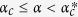, however, instead of being constant, the first-order critical point decreases and the second-order critical point increases as *α* decreases. The observation of such results may be mainly due to the small-world-like network structure^52^,^54^, which leads the transition showing a intermediate behavior. When *α* < *α*_*c*_, the hybrid phase transition vanishes and there is only a second-order phase transition with an increasing critical point as the decreasing of *α*. When *α* goes to negative infinity, where all long-range links are highly localized, the spatial networks degenerates into regular lattices and the transition is of second-order. In this way, we give a possible explanation of the emergence of these phase transition phenomena.

Moreover, our results are, to some extent, relevant to the control of information spreading. For example, when *α* ≥ *α*_*c*_, if we would like to make as many people as possible to know the information, the optimal choice of the fraction of initially informed people should be *p*_*c*1_, since larger initially informed population provides no more benefit but requires higher cost as shown in Fig. 2a. Therefore, the results help us in better understanding the self-organization of spatial structure of online social networks, in terms of the effective function for information spreading. Besides, our work can possibly find applications in studying the diffusion of virus spread in other spatial networks such as ad hoc, wireless sensor networks and epidemiological graphs. However, in the context of a numerical study, it is really hard to tell whether the critical value 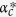 is exactly −1 and whether the critical points are completely independent of *α* when 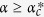. In addition, how does the transition depend on the spreading processes and what kind of transitions does it belong to for real social networks are still open questions. Hence, we expect to verify our findings in an analytical way and based on other generalized network models, including spatially constrained Erdös-Rényi networks^55^, network of networks (NON)^56^, multiplex networks^57^ and real social networks. Besides, to assign each node one long-range link is a high-cost strategy when generating artificial spatial social networks, we leave individualized number of long-range links associated with each node and partial spatial embedding as future works.

## Methods

To numerically implement the spatial scaling *α* when generating Kleinberg’s spatial networks, we add undirected long-range links to a 2-dimensional periodic square lattice in a smart way as follows. First, a random length *r* between 2 and *L*/2 is generated with probability 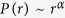, which ensures the scaling in advance. Second, random segmentations of length *r* to Δ*x* and Δ*y* with the only constraint that 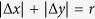 are done to determine candidate nodes, where Δ*x* and Δ*y* are both integers. Namely, for an uncoupled node *i* with coordinates (*x*, *y*), named target node, all candidate nodes are these with coordinates (*x* + Δ*x*, *y* + Δ*y*) such that 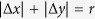. The above procedure ensures all candidate nodes at distance *r* from the target node *i* are uniformly distributed. Hence, we can randomly choose an uncoupled candidate node (i.e., a node without any long-range link) to link with target node *i*. Be noted that, on a large system, a finite fraction of the nodes will have all candidate nodes already connected when *α* is very small. To deal with this problem, we additionally adopt an alternate procedure referring to the distance coarse graining procedure^58^, in which we randomly choose an uncoupled nearest neighbor node of these candidate nodes until the linking is accomplished. We repeat such procedure for the rest uncoupled nodes until each node of the network has one undirected long-range link such that the degree of each node is exactly 5.

## Additional Information

**How to cite this article**: Gao, J. *et al.* Bootstrap percolation on spatial networks. *Sci. Rep.* **5**, 14662; doi: 10.1038/srep14662 (2015).

## Supplementary Material

Supplementary Information
