# Supplementary material for: Bootstrap percolation on spatial networks
Source: Sci Rep. 2015 Oct 1;5:14662. doi: 10.1038/srep14662 (PMC4589777; doi:10.1038/srep14662)
Supplement: Supplementary Information [file srep14662-s1.pdf]

Bootstrap percolation on spatial networks

Jian Gao[1], **Tao Zhou**[1,2] **& Yanqing Hu**[3,4,*]
[1] CompleX Lab, Web Sciences Center, University of Electronic Science and Technology of China, Chengdu 611731, China.
[2] Big Data Research Center, University of Electronic Science and Technology of China, Chengdu 611731, China.
[3] School of Mathematics, Southwest Jiaotong University, Chengdu 610031, China.
[4] School of Information Science and Technology, Sun Yat-sen University, Guangzhou 510006, China.
[*] E-mail: yanqing.hu.sc@gmail.com

Supplementary Information

1. S_{gc} for spatial networks

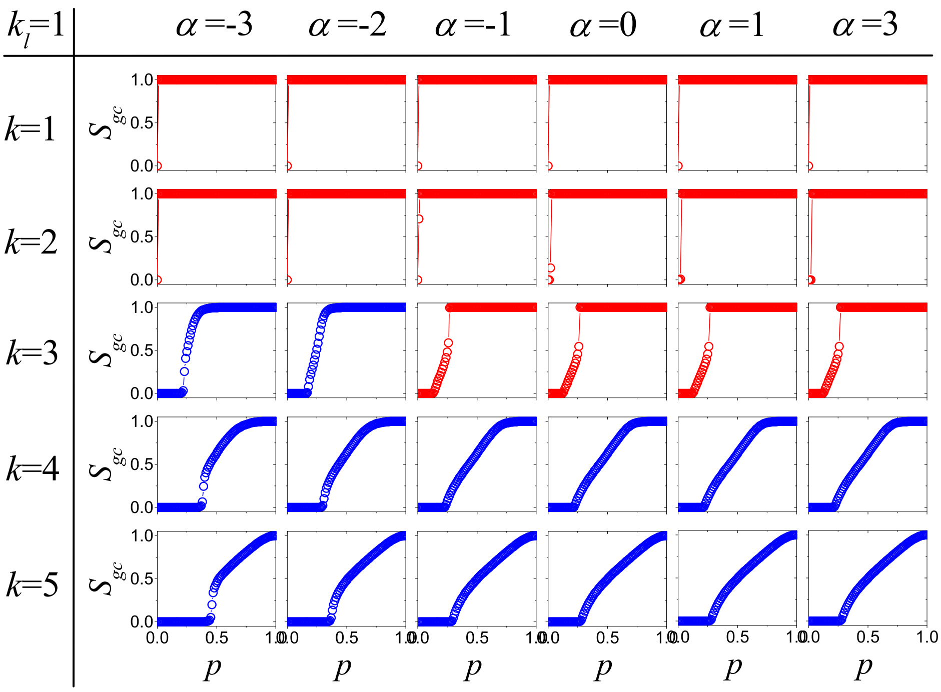

Figure S1. S_{gc} as a function of p after bootstrap percolation on undirected Kleinberg's spatial networks with $k_l = 1$ in parameter spaces (k, α). Red and blue curves are correspond to the present of a double phase transition (or a first-order phase transition in the trivial cases where $p_{c1} \approx 0$) and a second-order phase transition, respectively. Results are averaged over 1000 realizations with fixed network size $L = 400$.

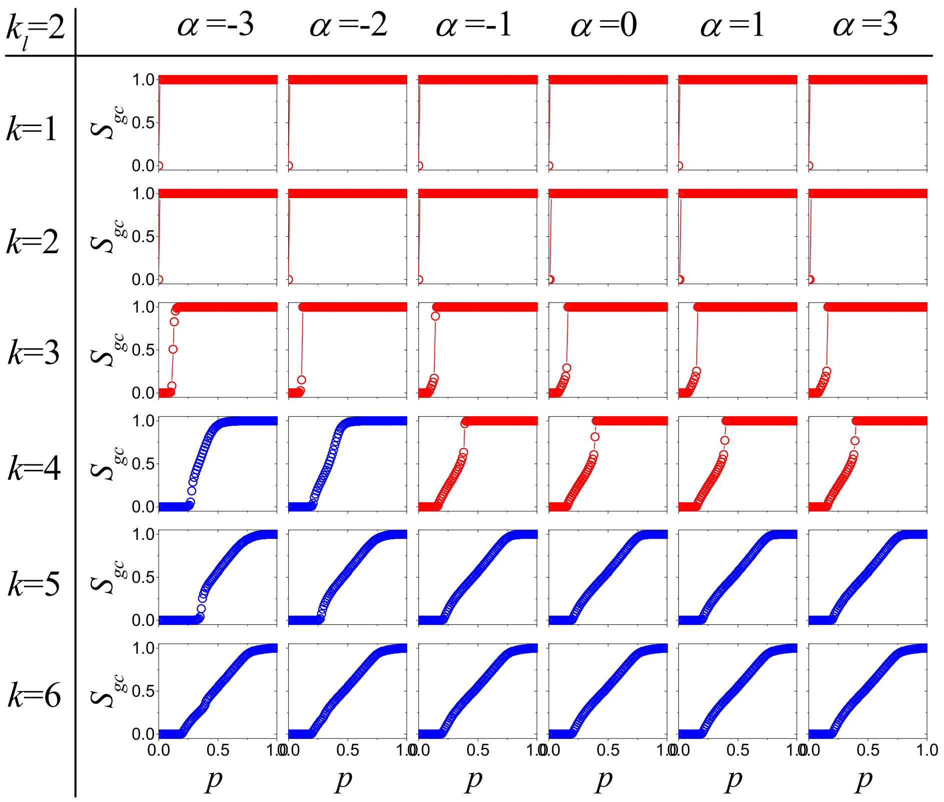

Figure S2. S_{gc} as a function of p after bootstrap percolation on undirected Kleinberg's spatial networks with $k_l = 2$ in parameter spaces (k, α). Red and blue curves are correspond to the present of a double phase transition (or a first-order phase transition in the trivial cases where $p_{c1} \approx 0$) and a second-order phase transition, respectively. Results are averaged over 1000 realizations with fixed network size $L = 400$.

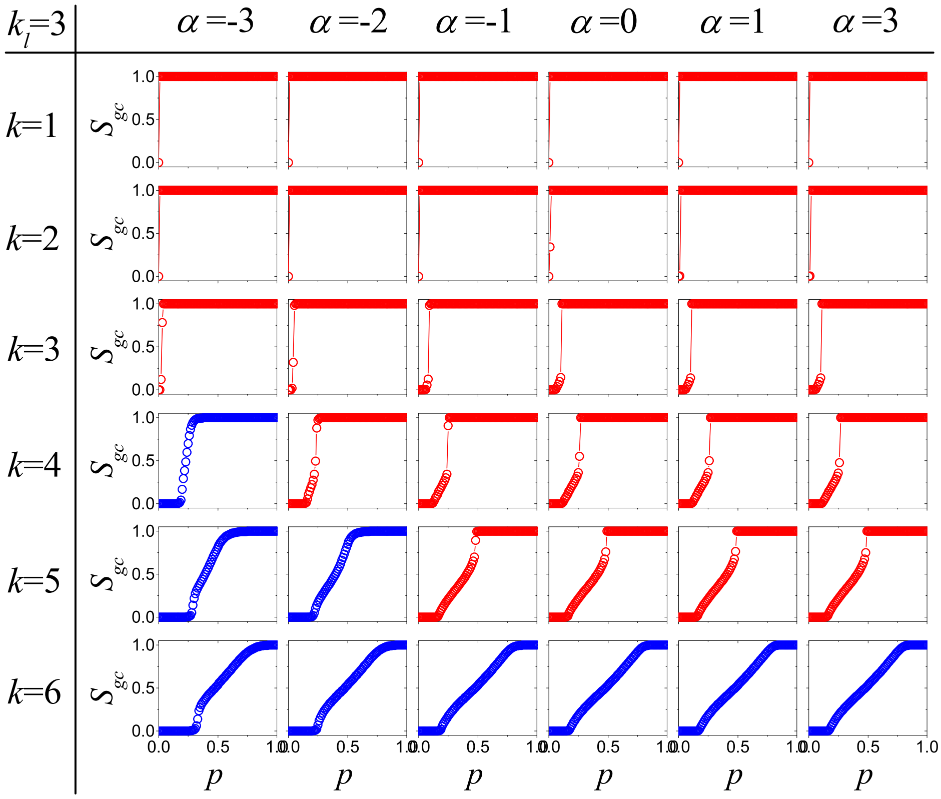

Figure S3. S_{gc} as a function of p after bootstrap percolation on undirected Kleinberg's spatial networks with $k_l = 3$ in parameter spaces (k, α). Red and blue curves are correspond to the present of a double phase transition (or a first-order phase transition in the trivial cases where $p_{c1} \approx 0$) and a second-order phase transition, respectively. Results are averaged over 1000 realizations with fixed network size $L = 400$.

2. S_{gc} for directed Kleinberg's spatial networks

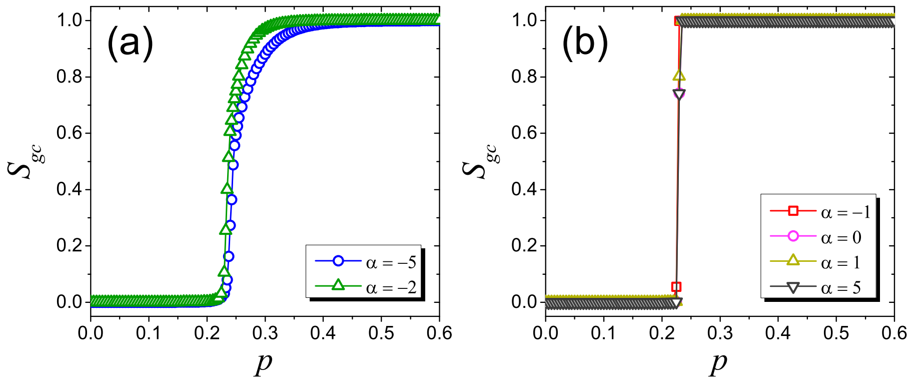

Figure S4. S_{gc} as a function of p after bootstrap percolation on directed Kleinberg's spatial networks in parameter spaces $(k, k_l) = (3, 1)$. Results are averaged over 1000 realizations with fixed network size $L = 400$.

3. Analysis on effects of boundary conditions

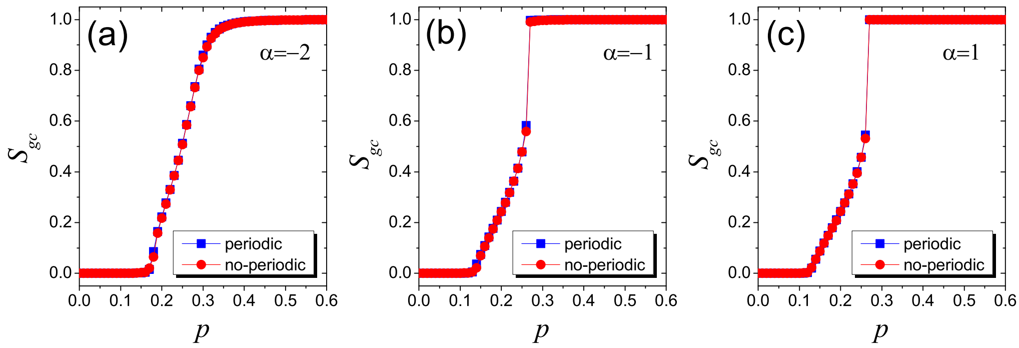

Figure S5. S_{gc} as a function of p after bootstrap percolation on undirected Kleinberg's spatial networks based on square lattice with or without periodic boundary conditions in parameter spaces $(k, k_l) = (3, 1)$. Results are averaged over 1000 realizations with fixed network size $L = 400$.

4. Phase diagram for LR networks

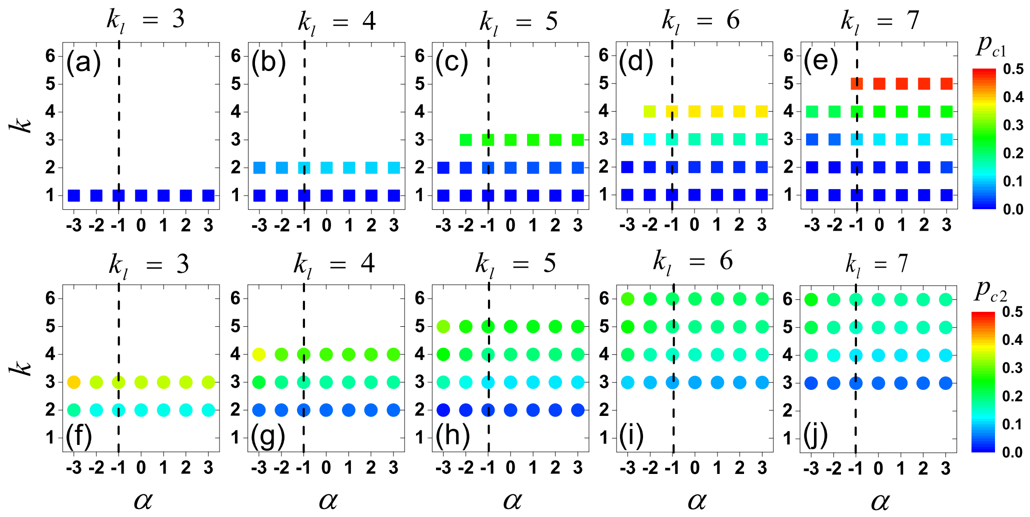

Figure S6. Phase diagram of bootstrap percolation on LR networks in parameter spaces (k, α, k_l). The color of data points in (a), (b), (c), (d) and (e) marks the value of p_{c1}, where there is a hybrid phase transition (or a first-order phase transition in the trivial cases where $p_{c1} \approx 0$), and the color of data points in (f), (g), (h), (i) and (j) marks the value of p_{c2}, where the transition is of second-order. Blank areas stand for the absent of the corresponding phase transitions. Separated by the vertical dash line $\alpha = -1$, on the right side, the color of data points is nearly unchanged for the same parameter k, meaning that the values of p_{c1} and p_{c2} are almost invariant. $\alpha_c \approx -1$ is found to be a robust critical value, above which the critical points for the double phase transition are almost constant. When $\alpha < -1$, p_{c1} decreases and p_{c2} increases as α decreases. Results are averaged over 1000 realizations with fixed network size $L = 400$.

5. S_{gc} for LR networks

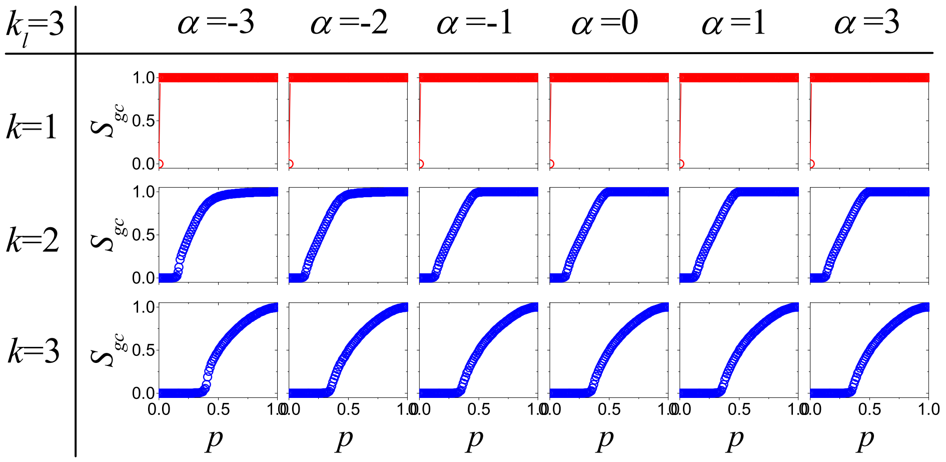

Figure S7. S_{gc} as a function of p after bootstrap percolation on LR networks with $k_l = 3$ in parameter spaces (k, α). Red and blue curves are correspond to the present of a double phase transition (or a first-order phase transition in the trivial cases where $p_{c1} \approx 0$) and a second-order phase, respectively. Results are averaged over 1000 realizations with fixed network size $L = 400$.

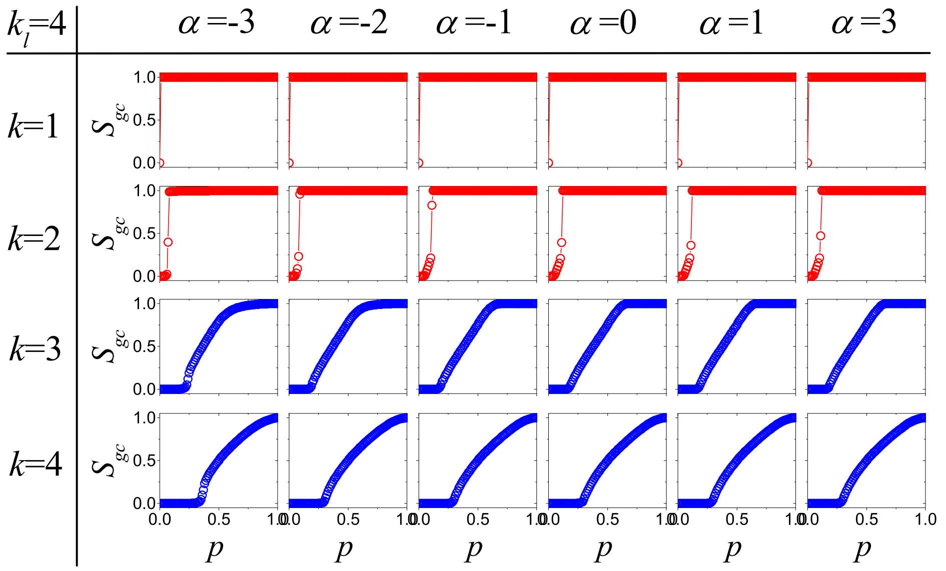

Figure S8. S_{gc} as a function of p after bootstrap percolation on LR networks with $k_l = 4$ in parameter spaces (k, α). Red and blue curves are correspond to the present of a double phase transition (or a first-order phase transition in the trivial cases where $p_{c1} \approx 0$) and a second-order phase, respectively. Results are averaged over 1000 realizations with fixed network size $L = 400$.

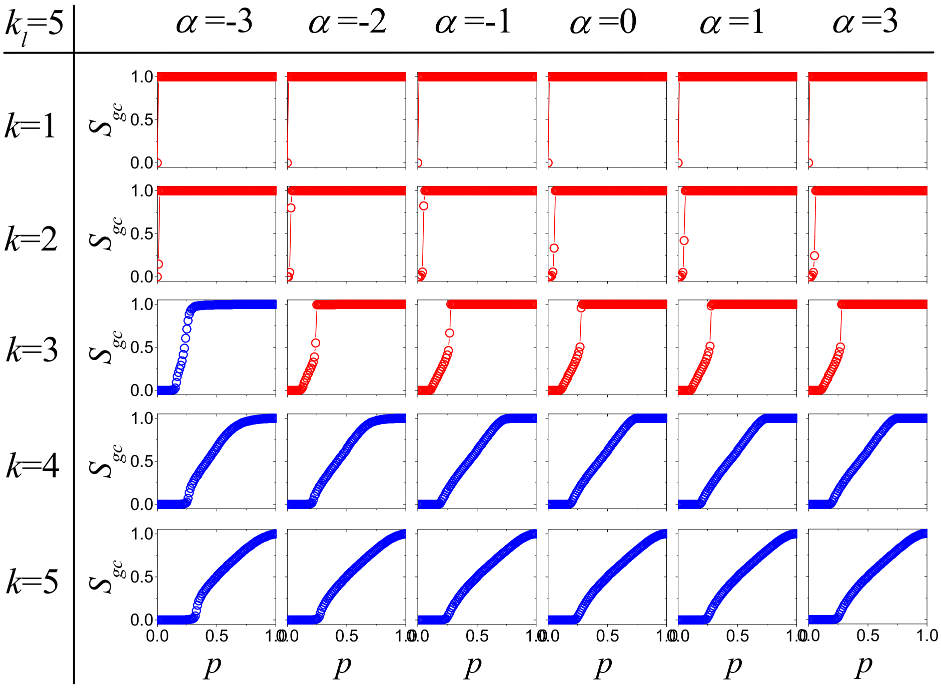

Figure S9. S_{gc} as a function of p after bootstrap percolation on LR networks with $k_l = 5$ in parameter spaces (k, α). Red and blue curves are correspond to the present of a double phase transition (or a first-order phase transition in the trivial cases where $p_{c1} \approx 0$) and a second-order phase, respectively. Results are averaged over 1000 realizations with fixed network size $L = 400$.

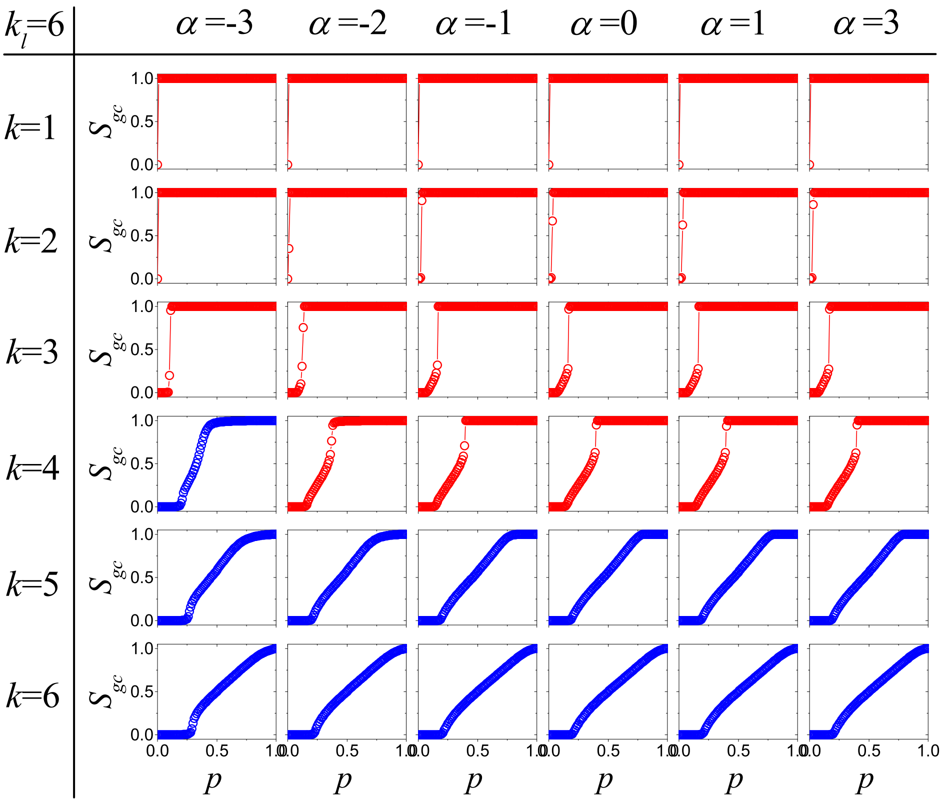

Figure S10. S_{gc} as a function of p after bootstrap percolation on LR networks with $k_l = 6$ in parameter spaces (k, α). Red and blue curves are correspond to the present of a double phase transition (or a first-order phase transition in the trivial cases where $p_{c1} \approx 0$) and a second-order phase, respectively. Results are averaged over 1000 realizations with fixed network size $L = 400$.

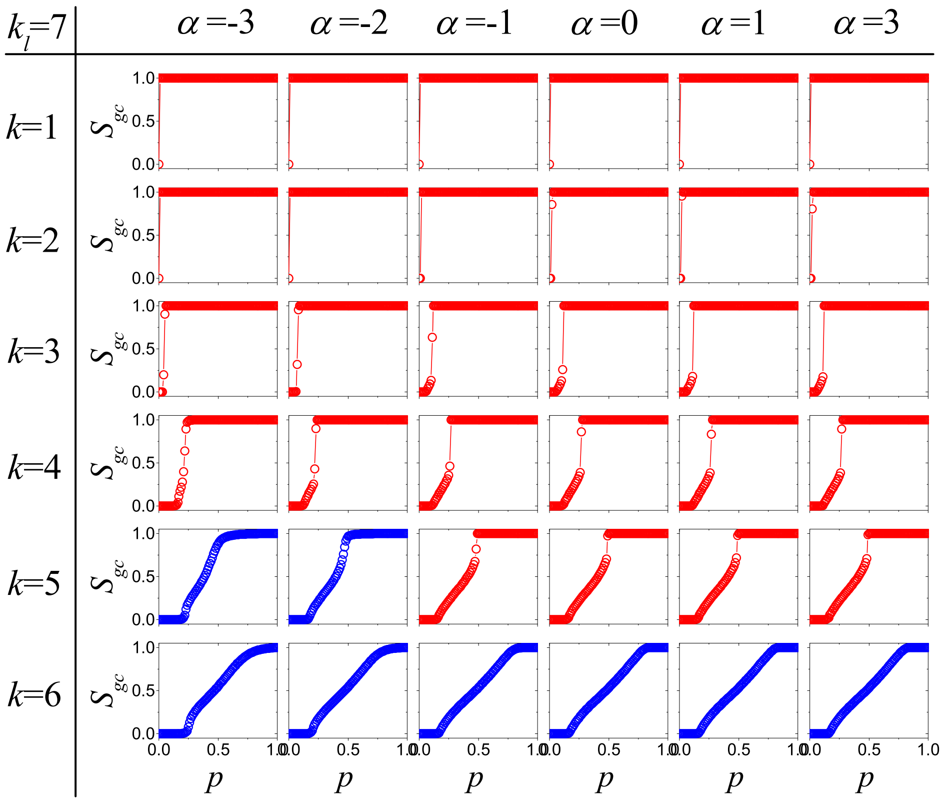

Figure S11. S_{gc} as a function of p after bootstrap percolation on LR networks with $k_l = 7$ in parameter spaces (k, α). Red and blue curves are correspond to the present of a double phase transition (or a first-order phase transition in the trivial cases where $p_{c1} \approx 0$) and a second-order phase, respectively. Results are averaged over 1000 realizations with fixed network size $L = 400$.
